# Development of Mortars That Use Recycled Aggregates from a Sodium Silicate Process and the Influence of Graphene Oxide as a Nano-Addition

**DOI:** 10.3390/ma16227167

**Published:** 2023-11-15

**Authors:** Jaime D. Ruiz Martinez, Héctor Cifuentes, José D. Rios, Pilar Ariza, Carlos Leiva

**Affiliations:** 1 Department of Chemical and Environmental Engineering, Escuela Superior de Ingenieros, Universidad de Sevilla, 41092 Seville, Spain; jruiz3@us.es; 2 Department of Continuum Mechanics and Structural Analysis, Escuela Superior de Ingenieros, Universidad de Sevilla, 41092 Seville, Spain; bulte@us.es (H.C.); jdrios@us.es (J.D.R.); mpariza@us.es (P.A.)

**Keywords:** mortars, sodium silicate waste, graphene oxide, mechanical test, alkali–silica reaction, acid resistance

## Abstract

This research analyses how different cement mortars behave in terms of their physical and mechanical properties. Several components were necessary to make seven mixes of mortars, such as Portland cement, standard sand, and solid waste from a factory of sodium silicate, in addition to graphene oxide. Furthermore, graphene oxide (GO) was selected to reduce the micropores and increase the nanopores in the cement mortar. Hence, some tests were carried out to determine their density, humidity content, water absorption capacity, open void porosity, the alkali–silica reaction, as well as flexural and mechanical strength and acid resistance. Thus, standard-sand-manufactured mortars’ mechanical properties were proved to be slightly better than those manufactured with recycled waste; the mortars with this recycled aggregate presented problems of alkali–silica reaction. In addition, GO (in a ratio GO/cement = 0.0003) performed as a filler, improving the mechanical properties (30%), alkali–silica (80%), and acid resistance

## 1. Introduction

Rapid urbanization and population expansion are predicted to raise the yearly production of waste to 3.4 billion tonnes over the next 30 years, up from 2010 million tonnes in 2016. According to a new World Bank estimate [1], worldwide trash creation is anticipated to increase by 70% by 2050 compared to present levels. This research underlines the importance of solid waste management in creating sustainable, healthy, and inclusive cities and communities. However, this is frequently disregarded, particularly in low-income countries. While more than a third of waste in rich countries is recovered through recycling and composting, only 4% of waste in underdeveloped countries is recycled.

The construction industry consumes many resources and materials, making it a sector with massive potential for utilizing waste materials created by its activities, as well as those generated by other industries. The reuse of such materials in cement-based materials not only reduces the demand for landfill capacity, but also reduces the need for raw material extraction [2]. Concrete recycling is a growing means of reusing the debris left over after concrete constructions are demolished or restored, giving a method for ensuring the long-term growth of these resources [3]. The most common sources of recycled concrete aggregates are those from construction and demolition waste, and from the precast industry [4,5,6,7], seashells [8], recycled brick powder [9,10], biomass and coal bottom ashes [11,12], and air-cooled blast furnace slag [13].

Even though their usage in structural concrete has been shown to have a favourable environmental impact [14], various studies have revealed particular concerns with the new concrete’s fresh and hardened properties. Furthermore, specific recycled concrete aggregates may include high concentrations of certain compounds (chlorides, alkali–silica interaction, sulphates, and heavy metals), which may affect their durability [15].

Sodium silicate is an inorganic chemical compound that can be produced from the combination of high-purity silica sand and sodium hydroxide. Sodium silicates are used in detergency, pneumatic, construction, food, paints and varnishes, agriculture, industrial and wastewater treatment, paper, rubber, and pharmaceutical industries, among others [16]. The wide range of physical and chemical properties of silicates is due to their wide range of possible compositions [17]. The silica and unreacted sodium hydroxide are part of the waste. Sodium silicate waste is dumped mainly in an open landfill [9].

Developing new solutions for the building industry indicates a promising future. The use of sodium silicate waste can be considered as a reactive aggregate from an alkali–silica reaction. There are several procedures to mitigate alkali–silica reaction: (a) the use of supplementary cementitious materials (e.g., ground granulated blast furnace slags and fly ashes) [18], (b) reducing the number of alkalis in the pore solution, [19] and (c) lithium chemical additions (e.g., lithium nitrate) [20].

The effect of the alkali–silica reactions could be reduced by utilizing nanoscale modifying agents to regulate concrete’s fresh and hardened properties, hence modifying the cement structure [21,22]. Many groundbreaking advances in nanotechnology have occurred in recent decades, demonstrating that matter may be manipulated and controlled at the nanoscale, even at the molecular and atomic levels [23].

Nanomaterial advancements have created tremendous opportunities to change the microstructure at the nanoscale and improve cement-based materials’ performance. The reinforcing mechanism depends on their shape and size, surface texture, interfacial bond strength (i.e., fiber/matrix interaction), crack control and recovery, and energy dissipation. In general, cracks are initiated within cementitious materials at the nanoscale [13]. The nanocracks then grow into micro- and macrocracks, which adversely affect the mechanical and durability properties [24]. Graphene oxide, silica, titanium oxide (TiO_2_), iron (Fe_2_O_3_), alumina oxide (Al_2_O_3_), CuO, calcium carbonate, ZnO_2_, and ZrO_2_ have been used during the last years in cement and concrete [25,26,27,28,29].

Graphene oxide (GO) has attracted the interest of nano-reinforcement in cement-based materials because of its remarkable mechanical capabilities, active functional groups, large specific area, and high thermal conductivity. The performance of GO-cement materials has been evaluated to transform traditional cement materials into smart, robust, and long-durable materials [30]. Graphene oxide is a thin layer of oxidised carbon composed of a single, dense layer of carbon atoms linked together in a hexagonal honeycomb lattice [31,32]. A GO sheet is a graphene derivative that consists of a hexagonal carbon network with functional groups such as hydroxyl (-OH), epoxide, carboxyl (-COOH), and carbonyl (=O) [33]. Because of these oxygen-containing functional groups, GO may easily produce stable water dispersions in the presence of polycarboxylate-based superplasticisers, [34] and may be more suitable for modifying all matrix properties. GO plays a core role in reducing the porosity of the cement matrix during curing the stage. Small amounts of GO, as little as 0.05%wt, increase compressive strength by 15–33% and flexural strength by 41–59% [35,36,37]. There is currently disagreement between the results reported by various researchers on GO-reinforced cement products because other studies [38,39] have found no improvements or even disadvantages [36,37,40]. Discrepancies in results may be caused by significant differences between studies. When previous studies are considered, three important factors can be observed to explain this controversy: (a) the different particle size of GO after ultrasonication; (b) if nanomaterials lack proper dispersion, they produce defects in the cement matrix, degrading various properties; and (c) the different porosity of the matrix to which GO is added (which depends on the water/cement ratio and the particle size of materials) [36,37,38,39,40].

The significance of this research is to investigate the effects of sodium silicate waste as fine aggregate and the effect of adding GO, analyzing the physical, mechanical, and durability properties of the mortars and environmental behavior through the analysis of the leaching of heavy metals of the waste.

## 2. Materials and Methods

### 2.1. Materials

**Figure 1:**
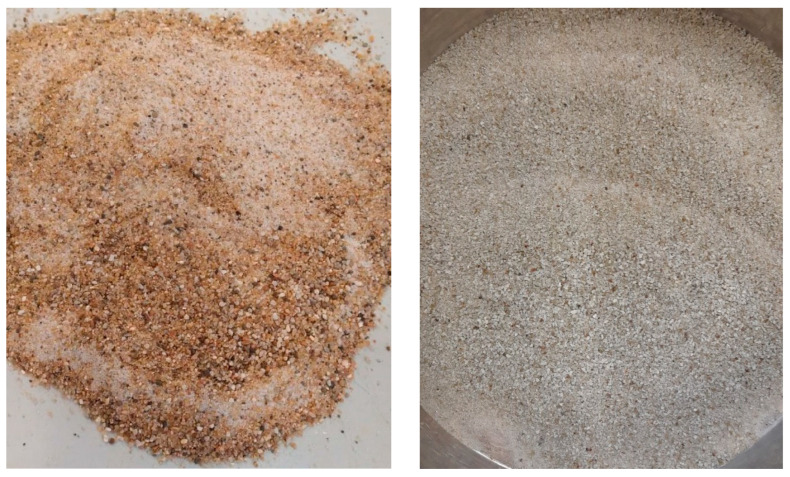
Standard sand (**left**) and recycled sand (**right**).

The cement used was Portland Valderrivas Cement CEM II/B-L 32.5 N, according to EN 197-1 [41]. A superplasticiser (20HE) from Sika Company was used. According to EN 196-1 [42], natural sand was used. Recycled aggregate came from a sodium silicate process from the south of Spain. Figure 1 shows the natural and recycled aggregates. At first glance, their main difference is the color; standard sand is yellow, and recycled sand is greyish.

Table 1 shows the major chemical composition of natural and recycled aggregates.

As observed in Table 1, silicon is the main element in both sands. Sodium is the other component that stands out in the recycled sand’s composition since it was previously attacked with sodium hydroxide. It could produce an alkali–silica reaction since most of the alkaline compounds in the waste come from the NaOH used and do not react during the sodium silicate process. Additionally, the effect of GO in the control of alkali–silica reaction was studied.

SO_3_ content is lower than the maximum limit (0.2%wt) established for any mortar aggregate in EN 13139:2003 [43]. The loss on ignition at 950 °C of recycled and natural aggregates is lower than 3%wt, a limit established by EN 13139:2003 [43] for air-cooled slags as fine aggregates. Specific gravity was determined using a pycnometer method, and the specific gravity of the recycled aggregate is 35% lower than natural, which indicates that the porosity is greater in recycled aggregate than in natural aggregates.

Table 2 shows the minority chemical composition of recycled aggregate. Between minor chemical components of recycled sand, lanthanum and yttrium could be found. They are considered “rare-earth elements”, expensive items that have increased their prices even more in recent years because their exportation has been diminished due to environmental reasons [44]. Other minor chemical components in the recycled sand, such as Cr, Pb, Ni, V, and Ba, are considered heavy metals, and it is necessary to analyse their leaching behaviour to recycle them as a construction material component.

**Figure 2:**
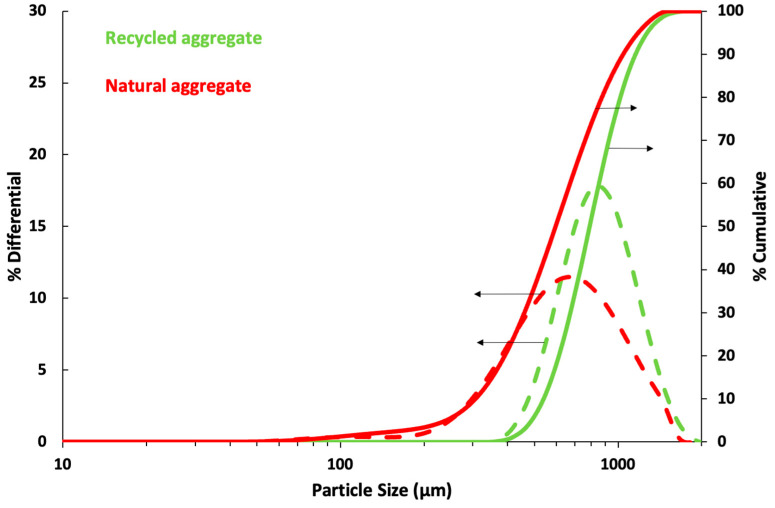
Particle size distribution of standard and recycled sand.

Particle size distribution of both aggregates, standard and recycled sand, can be seen in Figure 2. It has been measured using a Saturn DigiSizer II Particle Size Analyser. Cumulative per cent is represented by continuous lines, while dotted lines represent incremental per cent.

Standard and recycled aggregate distributions are both similar. Natural aggregates present a range between 0.1 and 1.5 mm and recycled between 0.4 and 1.5 mm. The average particle size of natural sand is 620 µm, while recycled is 830 µm.

**Figure 3:**
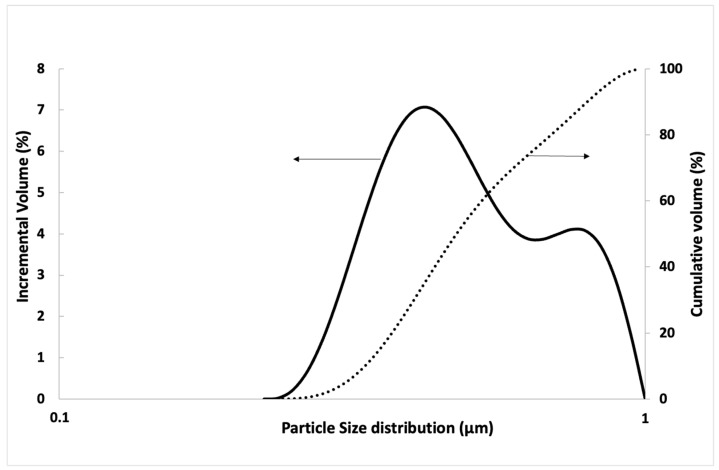
Particle size distribution of graphene oxide.

The GO solution was obtained from Graphenea and had a concentration of 4 g/L. However, the GO distribution is represented in another figure (Figure 3) due to its big size difference compared to the others. Particle size distribution of GO was determined after the following process: stirring for 24 h, followed by sonication for 30 min with a 360 W ultrasonic machine using the same GO/water used in this work and with the same superplasticiser dosage. Cumulative per cent is represented by continuous lines, while incremental per cent by dotted lines. Figure 3 shows that the size of the GO is less than 1 μm; GO requires a previous ultrasonication process to achieve a nanometric size.

### 2.2. Mix Design and Preparation of Mortars

Preparation of mortars has been carried out according to the specifications in EN 12390-2 [45]. Seven different kinds of mortars, seen in Table 3, were made to compare the influence of every material. The ratio of cement/aggregate is 1:3.

First, mixing different solid components using a laboratory kneader for 3 min was necessary. The water temperature, approximately ambient, was 20 °C. To obtain a better particle dispersion of graphene oxide in water, the mix of water, graphene oxide, and superplasticiser was previously agitated for 24 h and ultra-sonicated for 30 min in a bath sonication to produce stable nanomaterial suspension before mixing with the solids.

After adding the water solution to the solid mix, they were mixed for 5 min; a homogenous paste was formed. The next step was to shape the paste by employing some 16 cm × 4 cm × 4 cm-parallelepiped-shaped moulds and 4 cm height and 3.4 cm diameter cylinder moulds. They were vibrated for 2 min.

**Figure 4:**
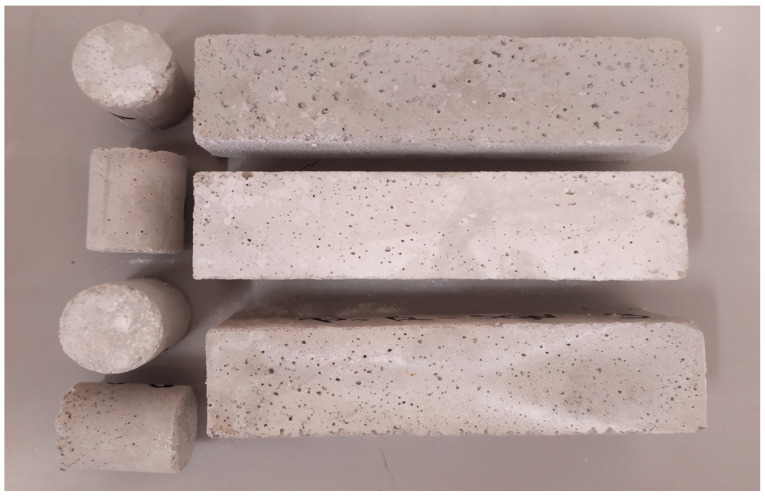
De-moulded samples (RA-0.37).

The samples were de-moulded after 24 h. All mortars were cured under water for 23 days at 20 °C and exposed to air for another four days. Figure 4 shows what parallelepipeds and cylinders looked like; as shown in Figure 4, efflorescence is not observed in the samples.

### 2.3. Methods

#### 2.3.1. Leaching Study

According to EN 13139:2003 [43] “The aggregates must not contain harmful materials in a susceptible quantity that may affect the durability or surface properties of the mortar to which they are incorporated”. The raw materials in any industrial process may contain very low levels of heavy metals. However, their wastes may be enriched, and the reuse of waste may present problems from the point of view of heavy metal leaching. Some European countries, such as Italy [46] and Portugal [47], establish maximum limits for the leaching of heavy metals in waste that can be used as construction material to ensure people’s health. There is no national regulation about heavy metal leaching in construction materials in Spain, but there is a regional regulation (Cantabria) [48]. Hence, all these regulations require the EN 12457-4 test [49], with a water-to-waste ratio of 10 L/kg dry matter. Waste must present a particle size below 10 mm (as seen in Figure 2).

#### 2.3.2. Physical Properties

Mortar density was calculated by taking parallelepipeds and cylinders’ average weight and volume measurements. It is a fundamental property because it can affect mechanical ones, such as flexural and compressive strength. The following calculation was necessary to reach that result:

*ρ* = M/*V* (1)

where *ρ* is the density (kg/m^3^), M is the mass (kg), and *V* is the volume (m^3^). Five samples were tested for each composition.

The volume stability was determined using the Le Chatelier apparatus in accordance with EN 196-3 [50]. Three samples were tested for each composition.

To obtain water absorption capacity (*A*), the samples were weighed (*Wd*), and after that, they were left under water for 24 h. Then, they were removed and weighed (*Ws*).

(2) $$A\left(\%\right)=\frac{Ws-Wd}{Wd}\cdot 100$$

The open void ratio (*VR*) was calculated by relating the volume in the sample occupied by water (*Vw*) and the total volume of the sample itself (*V*). Thus, the water volume is obtained as (*Ws* − *Wd*)/*ρw*, where *ρw* is the water density. Equation (3) sums up the required calculation to obtain *VR*:

(3) $$VR=\frac{Vw}{V}=\frac{\left(Ws-Wd\right)}{\rho w}\cdot \frac{1}{V}$$

A porosimetry investigation was conducted. A Micromeritics Autopore IV mercury intrusion porosimeter was employed. The pore sizes measured ranged from 0.007 to 150 m. The samples utilised were in the form of pellets of around 5 mm in size, and they had to be dried in an oven at 105 °C until they attained a constant mass.

To analyse the morphology in the mortars, a scanning electronic microscopy (SEM) test using the FEI Teneo model was carried out to examine mortar fragments.

#### 2.3.3. Alkali–Silica Reaction Evaluation

Expansion measurements were taken under ASTM C 1260 [51]. Each mortar mix was cast into three 25 × 25 × 286 mm mortar bars and three different compositions: NA) cement/natural aggregate = 1/2.25; RC) cement/recycled aggregate = 1/2.25 and RC-GO) cement/recycled aggregate = 1/2.25 with GO/cement = 0.1/333.33. All the samples used a water/cement ratio = 0.47. Following the initial curing step, specimens were de-moulded and stored in water at 80 °C for 24 h. After that, the specimens were immersed in a 1 N NaOH solution at 80 °C to speed up the alkali–silica reaction and expansion. Expansion measurements were taken at two-day intervals.

#### 2.3.4. Mechanical Properties

Mechanical properties were tested experimentally 28 days after the sample was made, according to the Standard EN 1015-11 [52], using a Tinius Olsen-TO317EDG machine (Surrey, UK). A total of five 16 × 4 × 4 cm parallelepipeds of every mix were used to perform flexural tests. A total of six 8 × 4 × 4 cm parallelepipeds of every mix were used to perform the compressive strength test.

Based on a previous study [12,53], acid attack resistance was determined by submerging cylinders of 4 cm in height and 33 mm in diameter completely in 1 M sulphuric acid for 14 days. After this process, the compressive strength was determined and compared with the compressive strength of the samples in air for 14 days. Results were expressed as the acid variation according to:

(4) $$Acid\;variation\;\left(\%\right)=\frac{Ci-Cair}{Cair}\cdot 100$$

where *Ci* is the compressive strength (MPa) of mortars immersed in acid, and *Cair* is the compressive strength (MPa) of non-immersed mortars.

## 3. Results

### 3.1. Leaching Results

Table 4 compares standard and recycled sand leaching results to the limits stated for different parameters by the EU waste landfill directive [54] according to EN 12457-4 [49]. European Landfill Directive [54] defines three categories: hazardous, non-hazardous, and inert wastes; according to Table 4, recycled aggregate can be classified as inert waste. In Portugal, the Environment Agency [47] established that wastes can be recycled in construction materials when limits for inert waste are not exceeded, so recycled aggregate could be used as aggregate in mortars. In Italy, the incorporation of wastes into construction materials is regulated according to Ministerial Decree 186 limits [46]. Table 4 shows that standard and recycled sands could be considered inert waste, but the recycled sand does not satisfy the requirements legally established by the Italian limit for Pb. An amount of 38% of the Pb present in the waste is leached, exceeding the limits established by the Italian Decree 186. However, for the other heavy metals present in the waste according to Table 2 (Ba, Cr, Ni, Sr, and V), no more than 12% is leached. In Spain, there are no national regulations, but there are some regional regulations, such as Cantabria [48], which have established leaching limits according to these test results for the valorization of some wastes as construction materials. According to these limits, the recycled aggregate cannot be used due to the Pb limit, as the Italian limit.

### 3.2. Physical Properties

**Figure 5:**
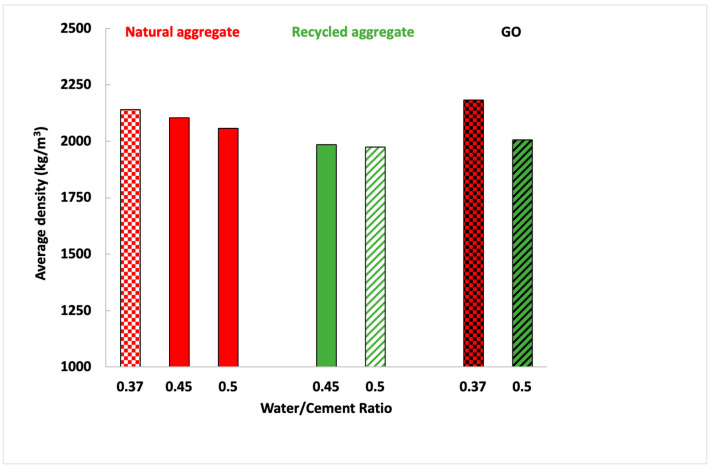
Density of different mortars.

Regarding the density, several factors have been analysed. Figure 5 shows the density difference between mortars manufactured with natural or recycled aggregates, different water ratios, and with or without GO addition.

**Figure 6:**
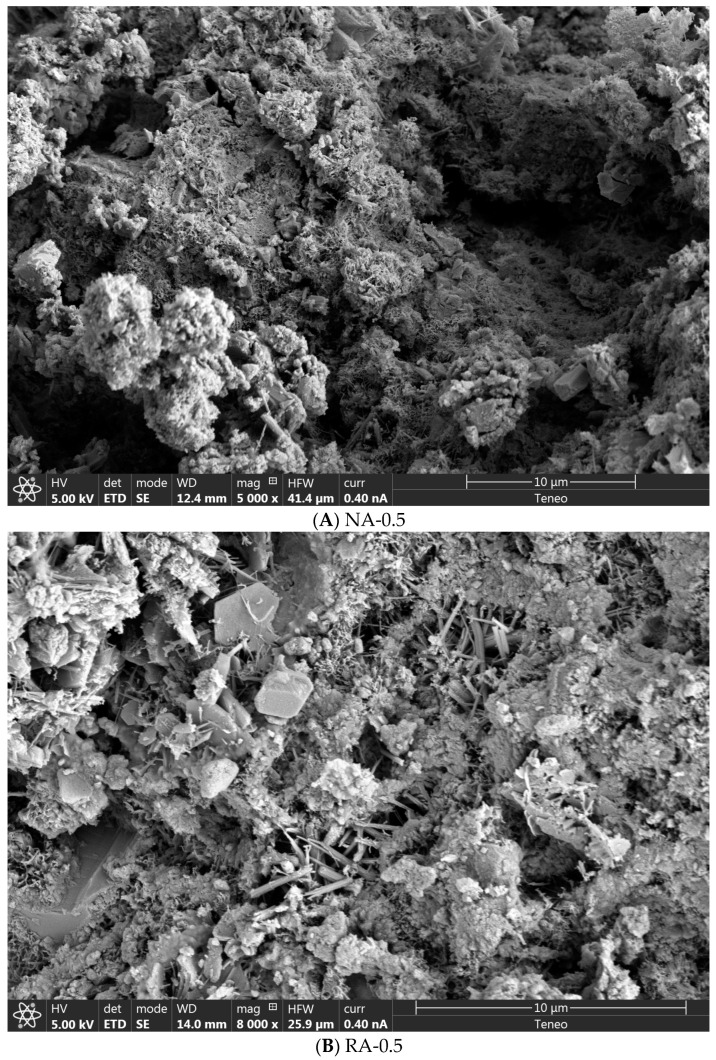

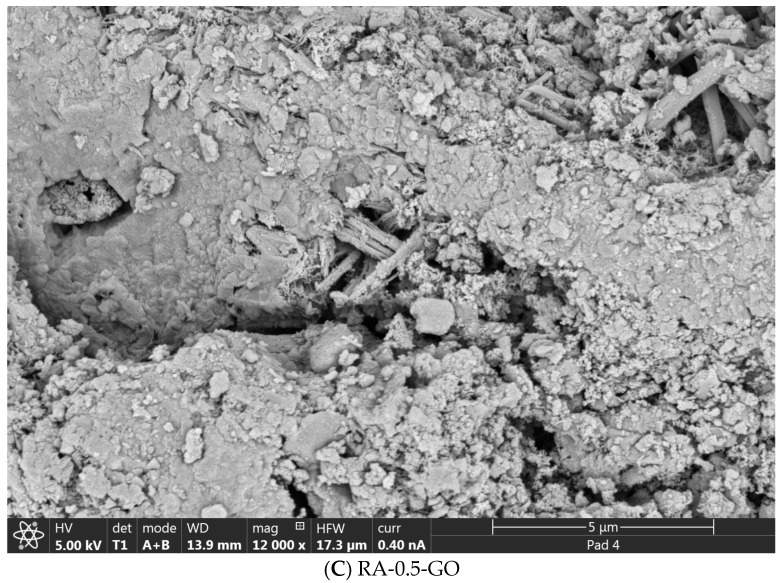
SEM of NA-0.5 (**A**), RA-0.5 (**B**), and RA-0.5-GO (**C**).

As can be seen, when a mortar is manufactured with a higher water/cement ratio, density becomes lower since there is a higher amount of water, and, consequently, the excess water is evaporated, and a higher number of pores are produced (Figure 6A,B). However, as could be observed in Figure 2, as recycled aggregate presented a slightly higher particle size distribution (Figure 2) and lower specific density (Table 2), the density of mortars manufactured with recycled sand was lower than those manufactured with the natural one.

**Figure 7:**
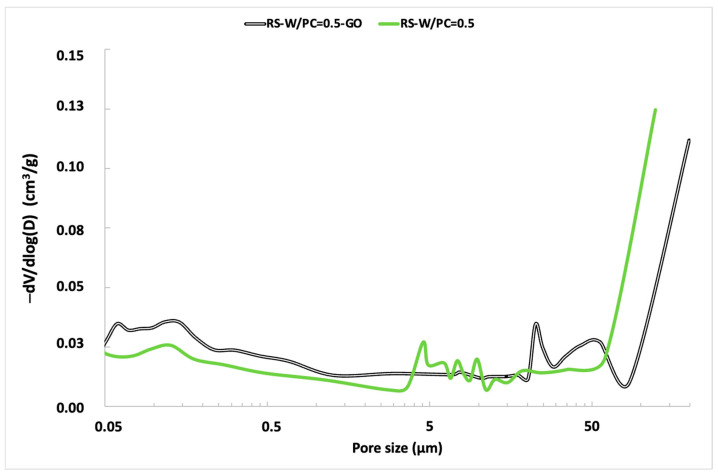
Mercury intrusion porosimeter results.

On the other hand, there is no density difference due to GO addition because the percentage of GO in the mix is inadequate, and it does not affect significantly. Nevertheless, the effect of GO on the pore size distribution was very significant (Figure 7). The number of large pores (higher than 50 μm) decreased, and a more significant number of pores between 10 and 50 μm appeared. The same occurred for those in the range between 0.05 and 1 μm; owing to the GO addition, the number of pores of that size was increased. By adding GO, large pores decreased and were divided into smaller pores of different sizes (Figure 6C).

Generally, SEM images of AR-0.5 depict common types of alkali–silica reaction products, which are foil-like crystals, rosette-like deposits, and hexagonal acicular crystals [55]. GO nanomaterials produce an interlocking effect, as seen in Figure 6C.

Volume stability of mortars experienced variations lower than 2 mm in all the cases. According to European standards [41], it must be less than 10 mm. The MgO content of cement and fine aggregate may have a negative impact on its volume stability [56]. Because of the low MgO content of the cement and sand, all of the samples in this research have volume stability lower than 2 mm.

However, the volume stability is required to be lower than 10 mm. A factor that may negatively influence the volume stability of cement with fly ash is the MgO content [56]. In this study, all the samples have a volume stability of 2 mm due to the low MgO content of the cement and sand (<4%).

Table 5 shows the average results of other physical properties, such as water absorption capacity and open void porosity. As the water/cement ratio increases, these properties also increase because excess water evaporates and produces more pores [25]. The open void porosity of mortar with recycled aggregates is slightly higher than that of natural aggregates because they present a low specific density (Table 1) and a slightly lower particle size (Figure 2). Open porosity and water absorption capacity decrease, especially when GO is added, since many large pores are divided by GO, decreasing the number of large pores (Figure 6) where water can penetrate and, therefore, decreasing the open porosity.

### 3.3. Alkali–Silica Reaction

**Figure 8:**
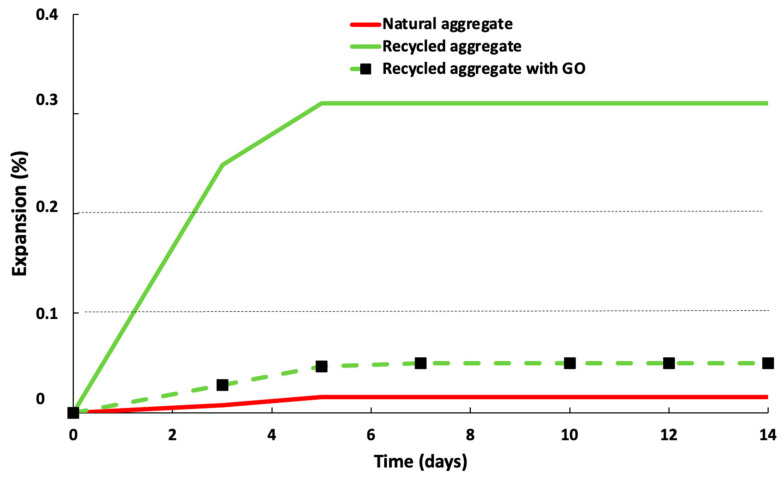
Expansion mortars according to ASTM C 1260 test.

Figure 8 depicts if the growth rate observed during the alkali–silica test at 14 days is 0.1, 0.2%, or greater. The aggregate is reactive to the alkaline-silica reaction, whereas growth of less than 0.1% at 14 days suggests a highly innocuous aggregate. Intermediate 14-day expansions of 0.1% to 0.2% indicate a population that may be damaged by alkali–silica reaction, but this has to be investigated further (28 days). The addition of GO diminished the porosity (especially the open porosity), obstructing the external alkali ions from attacking the aggregate particles. Furthermore, according to previous studies [21,25,30], GO presents a larger surface area, remodelling and refining the C–S–H gels around the interfacial transition zone and changing the chemical composition of the pore solution. It prevents the alkali–silica reaction due to internal alkali ions. By increasing the pore structure, bridging fractures, and accelerating the hydration process, GO may be able to resist alkali–silica reaction cracking via chemical and mechanical processes [57].

### 3.4. Mechanical Properties

#### 3.4.1. Flexural Strength

**Figure 9:**
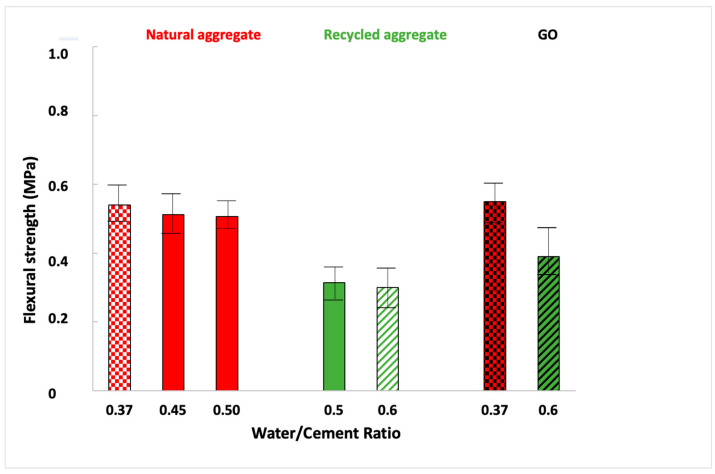
Flexural strength results.

Figure 9 shows that flexural strength diminished when mortars were manufactured with a more significant amount of water. When the excess water added was evaporated during the curing process, porosity was increased, as well as the preferential breaking paths in the material [58]. Thus, it proves that this property follows the same trend as density.

On the other hand, comparing the influence of each aggregate, a significant difference was observed: the recycled aggregate mortar presents a low flexural strength. GO addition led to a slight improvement when mortars were in both sands. The bond strength between GO and the mortar matrix depends on multiple factors, such as GO dosage, ultrasonication process, matrix composition, and water/cement ratio [59] As the ultrasonication process seems adequate (Figure 3), in this case, the matrix composition and water/cement ratio used decreased the density of the mortar matrix, decreasing the quality of GO dispersion (see Table 1) by blocking the free movement of GO [59]. Recent studies [60] showed that flexural strength increased when another industrial waste material was employed as a substitute for fine aggregates, such as quarry dust or limestone dust, with a water–cement ratio of 0.52, like the one in this research. Flexural strength obtained using mortars partially made of quarry or limestone was up to 25% higher than those manufactured with only natural fine aggregate.

#### 3.4.2. Compressive Strength

**Figure 10:**
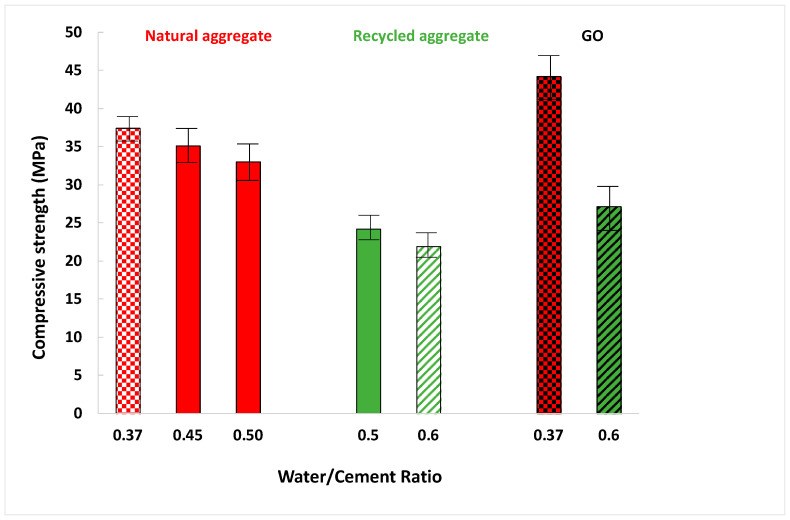
Compressive strength results.

Figure 10 depicts that compressive strength diminished when mortars were manufactured with a more significant amount of water. Thereby, it also proves that this property follows the same trend as density and flexural strength.

First, comparing the influence of both sands, it was registered that recycled sand presents 73% of the compressive strength with standard sand. Nevertheless, every mortar exceeds 17.2 MPa, a value set by Standard ASTM C270-02 [61] for mortar for masonry. On one hand, it is very usual that recycled mortars present lower compressive strength than standard sands [13,26]. On the other hand, other research [60] proved that compressive strength increases by around 20% when other wastes (quarry dust or limestone dust) are employed as substitutes for fine aggregate.

Second, mortars manufactured with GO had better compressive properties. Specifically, GO addition in sand mortars led to an improvement of approximately 18%. The increase in compressive strength with GO could be associated with its performance in mortar porosity, whereby the GO particles filled its internal pores and strengthened its internal structure, which improved compressive strength (Figure 6C and Figure 8). Many earlier studies show that GO additions increase the compressive strength of mortars and concrete [21,25,30], and many others show that GO additions do not improve mechanical properties [62,63]. This divergence may be due to two different factors: (1) The different sizes of the GO used after sonication and the pore sizes of the matrix. If the GO particle size is much greater than that of the pores of the matrix, no reinforcement effect is produced and, therefore, no mechanical improvement is produced; (2) The degradation in compressive strength might be related to poor dispersion of GO particles, resulting in higher porosity [59].

#### 3.4.3. Acid Attack

**Figure 11:**
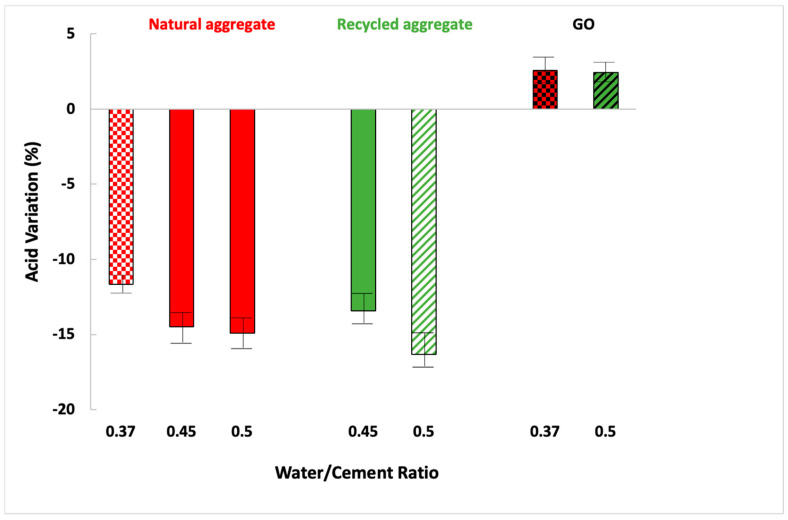
Compressive strength difference between acid-immersed and non-immersed mortars.

These results are presented as the compressive strength variation that a mortar underwent after being immersed in acid for 14 days compared to another that was not immersed during that time. Figure 11 shows compressive strength variation between immersed in acid and non-immersed mortars.

In general terms, compressive strength diminished after the acid attack when mortars were manufactured with a higher amount of water because its porosity was higher, increasing the water penetration and reducing the compressive strength. None of the sand-manufactured mortars improved their compressive strength after the acid attack because their open void porosity is higher (Table 5) and similar for both types of aggregates. Mortars manufactured with both types of sand presented a higher open void porosity (macro-pores connected to the superficial zone), increasing the acid penetration.

Because calcium hydroxide combines with sulphate ions, the acid always generates a white layer of gypsum. Furthermore, the reaction between gypsum and calcium aluminates in the cement matrix can yield ettringite [64]. Ettringite expands more than gypsum, resulting in fissures inside the matrix after an acid attack.

GO enhanced their compressive strength after the acid test (at the same water/cement ratio). GO causes the bigger pores to close, preventing acid from entering the matrix’s core (Table 5 and Figure 7). It is, however, not entirely free of pores. Small amounts of sulphate eventually enter the sample and react to generate a small quantity of ettringite, which favours the specimens’ strength [65].

**Figure 12:**
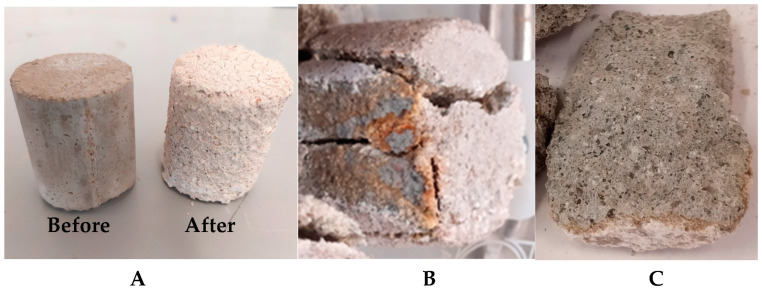
(**A**) Recycled sand mortar (RA-0.5) before and after being water- and acid-immersed (with white colour on its surface) (**B**) RA-0.5 after the acid test, and (**C**) sample inside (RA-0.5-GO) after the acid attack.

Figure 12A shows two samples made of recycled sand (RA-0.5), where the outside of the right one is white due to the formation of CaSO_4_·H_2_O during the acid attack. Figure 12B shows the inside of the attacked mortar (RA-0.5) after the compression test, where it can be seen that the acid has penetrated the interior area of the mortar through the pores [25]. Figure 12C shows what the inside of RA-0.5-GO looked like after the compression test, where the cementitious colour remained with some white dots because the acid had not penetrated in high quantities inside the sample.

## 4. Conclusions

To conclude this research, several points were established related to the physical and mechanical properties of the mortars because of their water/cement ratio, used sand, and GO presence.

- It was confirmed that mortars manufactured with less water (lower water/cement ratio) have better physical and mechanical properties because it led to a lower-porosity mortar, and a diminution of 3% of water increased the mechanical properties by 10%.
- From leaching behaviour, the waste can be classified as inert waste but presents a high Pb leaching, which prevents it from being used as a construction material according to some legislations for construction buildings (Italy and Cantabria).
- Moreover, natural sand mortars are slightly better than recycled ones since, natural sand possesses fewer internal pores and, consequently, higher density (10%) and higher flexural (60%) and compressive strength (38%) were obtained for standard sand.
- GO reduces the porosity of the mortar and it is an effective material in controlling the expansion of alkali–silica reaction, reducing the expansion by more than 80%. GO increases the mechanical properties (30% of the compressive strength of recycled mortars). GO improves the acid resistance, increasing the compressive strength after the acid attack compared to the values obtained before the attack.

## Figures and Tables

**Table 1:** Major chemical compositions.

Component (%)	Recycled Aggregate	Natural Aggregate	Portland Cement
Al_2_O_3_	-	0.76	5.74
CaO	-	0.13	60.89
Fe_2_O_3_	-	0.22	2.46
K_2_O	-	0.30	0.73
Na_2_O	4.93	0.05	0.36
SO_3_	-	0.02	1.11
SiO_2_	92.18	96.21	20.96
TiO_2_	-	0.12	0.28
Loss on ignition	2.46	0.31	5.20
Specific gravity (g/cm^3^)	1.73	2.62	3.15

**Table 2:** Minor chemical compositions of recycled sand (ppm).

**Ba**	**Cr**	**Ga**	**La**	**Mn**	**Mo**	**Nb**	**Ni**
12.5	39.5	1.2	7.6	39.0	1.1	1.0	5.6
**P**	**Pb**	**Sr**	**Ta**	**V**	**Y**	**Zr**	**F**
38.3	4.8	5.5	2.9	3.2	4.4	349	336

**Table 3:** Mortar mix design.

Material Mix Design	Cement (kg)	Natural Aggregate (kg)	Recycled Aggregate (kg)	Superplasticiser (kg)	Water (L)	Graphene Oxide (kg)
NA-0.37	333.3	999.9	-	42	123.3	-
NA-0.45				42	150.0	
NA-0.5				42	166.7	
NA-0.37-GO				42	123.3	0.10
RA-0.45		-	999.9	42	150.0	-
RA-0.5				42	166.7	
RA-0.5-GO				42		0.10

**Table 4:** Comparison of the leaching results (mg/kg).

	Recycled Sand (mg/kg)	Standard Sand (mg/kg)	Inert Waste [54] and Portuguese Limit [47]	Non-Hazardous Waste [54]	Italian Limit [46]	Cantabria Limits [48]
As	<0.01	≤0.01	0.5	2	0.5	0.5
Ba	0.25	0.82	20	100	10	20
Cd	<0.01	≤0.01	0.04	1	0.05	0.04
Co	<0.01	≤0.01	-	-	2.5	-
Cr	0.176	≤0.02	0.5	10	0.5	0.5
Cu	<0.1	≤0.1	2	50	0.5	2
Hg	≤0.005	≤0.005	0.01	0.2	0.01	0.01
Mo	<0.05	≤0.2	0.5	10	-	0.5
Ni	0.21	≤0.01	0.4	10	0.1	0.4
Pb	1.84	≤0.25	0.5	10	0.5	0.5
Sb	≤0.02	≤0.02	0.06	0.7	-	0.06
Se	<0.01	≤0.025	0.1	0.5	0.1	0.1
Sr	0.65	-	-	-	-	-
V	<0.1	≤0.1	-	-	2.5	-
Zn	<0.01	0.067	4	50	0.03	4

**Table 5:** Volume stability, water absorption capacity, and open void porosity results.

Mortar	Volume Stability (mm)	Water Absorption Capacity (%)	Open Void Porosity (%)
NA-0.37	<2	8.3 ± 0.3	27.1 ± 0.9
NA-0.45	<2	10.1 ± 0.4	30.3 ± 1.2
NA-0.5	<2	10.8 ± 0.4	32.8 ± 1.2
NA-0.37-GO	<2	4.94 ± 0.6	12.9 ± 0.6
RA-0.45	<2	12.5 ± 0.7	33.3 ± 1.2
RA-0.5	<2	15.9 ± 0.9	33.9 ± 0.5
RA-0.5-GO	<2	6.8 ± 0.2	14.2 ± 0.5

## Data Availability

Not applicable.
